# Cutaneous leishmaniasis control in Alta Verapaz (northern Guatemala): evaluating current efforts through stakeholders’ experiences

**DOI:** 10.1186/s40249-021-00842-3

**Published:** 2021-05-07

**Authors:** Renata Mendizábal-Cabrera, Isabel Pérez, Víctor Becerril Montekio, Freddy Pérez, Erick Durán, Mei L. Trueba

**Affiliations:** 1 Center for Health Studies (CHS), Universidad del Valle de Guatemala (UVG), 18 Avenida 11-95, zona 15, V.H.3, Guatemala City, Guatemala; 2 Instituto Nacional de Salud Pública (INSP), Cuernavaca, Mexico; 3 Communicable Diseases and Environmental Determinants of Health Department, Pan American Health Organization, Washington, DC USA; 4 Leishmaniasis Sub-Program, National Ministry of Health of Guatemala, Guatemala City, Guatemala; 5 Department of Global Health and Infection (GHI), Brighton and Sussex Medical School (BSMS), University of Sussex, Brighton, UK

**Keywords:** Cutaneous leishmaniasis, Control effort, Qualitative evaluation, Stakeholders’ experiences, Alta Verapaz, Guatemala

## Abstract

**Background:**

Cutaneous leishmaniasis (CL), endemic in Guatemala, mostly affects poor people living in the northern region. A national control program that includes surveillance, diagnose, and treatment offered free of cost by the Ministry of Health (MoH) has been in place since 2003. However, the incidence is increasing and treatment rates are not optimal, suggesting that current efforts are not being effective. This study aimed to understand barriers and facilitators of CL control in Guatemala as experienced and perceived by key stakeholders in order to comprehend what works well and does not and suggest evidence-informed interventions.

**Methods:**

The study was conducted in the Cobán municipality, the most endemic of Guatemala, situated in the Department of Alta Verapaz. Data were collected during May and June 2019 via focus groups and semi-structured interviews with key stakeholders, including local and national health personnel and residents of four communities of the endemic region. Thematic and content analysis of the collected data was conducted using NVIVO.

**Results:**

Three overarching issues hamper the effectiveness of current CL efforts: resource scarcity, treatment challenges, and knowledge-action gaps. Scarce economic resources from the MoH and community residents negatively impact incidence, detection of cases and treatment rates in that preventive action is insufficient and healthcare access is low. In addition, local health workers often lack specialized CL training and access to the national CL control guidelines. With regards to the population living in the study area, misunderstanding of disease causation, shame associated with CL lesions, treatment pain fear, and long (often uncertain) waiting times for diagnose and treatment negatively affect people’s willingness to seek help, treatment adherence, and their trust on the healthcare provided.

**Conclusions:**

Culturally sensitive CL preventive action must be developed. Given the scarce economic resources available for CL control in the country, the involvement of trained community health workers and the inclusion of thermotherapy as a treatment option is also advised. Other cost-effective actions include: ensuring all health workers receive CL training and have access to national CL control guidelines, improving national procurement system to avoid treatment shortages, and provision of motorized vehicles to increase active surveillance and treatment rates.

**Graphical abstract:**

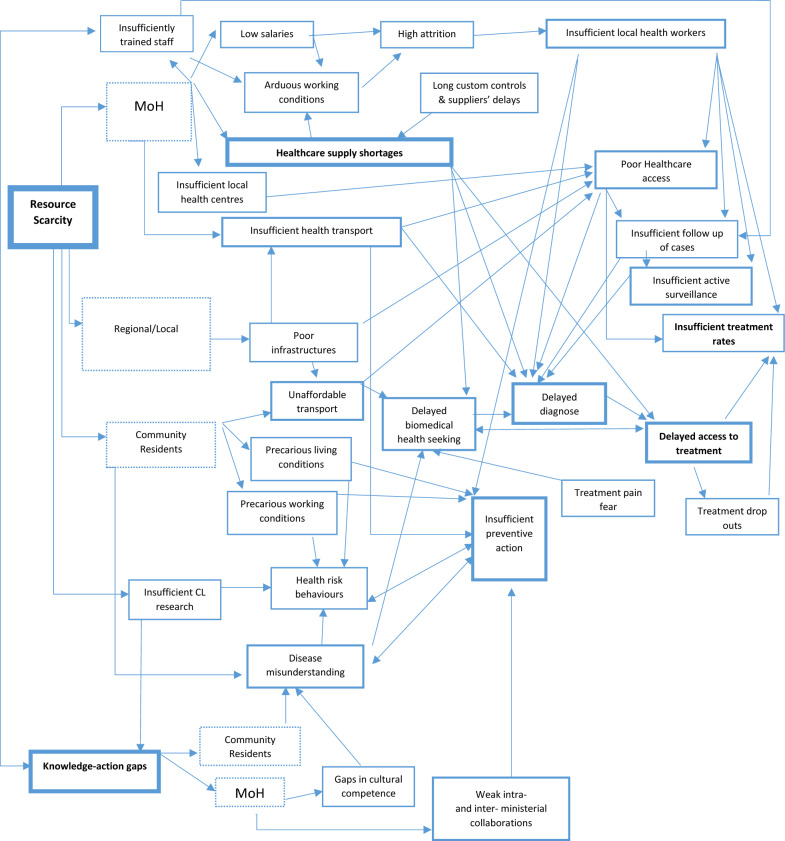

**Supplementary Information:**

The online version contains supplementary material available at 10.1186/s40249-021-00842-3.

## Background

Cutaneous leishmaniasis (CL), the most common form of leishmaniasis affecting humans, is a parasitic skin infection caused by the bite of *Leishmania* infected female sandflies. It is estimated that CL affects between 600 000 to 1 000 000 people per year worldwide; mostly in the Americas, the Mediterranean basin, the Middle East and Central Asia [1]. Generally causing disfiguring skin ulcers, injury and disability [2], this neglected tropical disease (NTD) is recurrent in poor rural regions and most vulnerable populations, often with difficult or no access to health services [3]. In Latin America it is present in 20 countries, being endemic in 18 of them [4].

Three million people are estimated to be at risk of contracting CL in Guatemala [5], mostly in the northern regions, where the disease is known as “*la chiclera*” because it was very common among gum collectors (*chicleros)* [6]. According to data provided by the *Sistema de Información Gerencial de Salud* (SIGSA), 1357 new cases were reported nationwide in 2019 (Additional file 1: Table S1). A total of 831 cases (61%) were diagnosed in Alta Verapaz, representing an incidence rate of 72.26 per 100 000 inhabitants (Additional file 1: Table S2). Our study was conducted in Cobán, the most endemic municipality of this northern department, where 200 cases were reported in 2018 and 190 in 2019.

CL can cause from a single small ulcer to large, multiple lesions generally located in exposed areas of the body. Most cases of CL in Guatemala are caused by *L. braziliensis* or *L. mexicana*. Infections caused by *L. braziliensis* progress rapidly, rarely resolve without specific therapy, and often respond rapidly to treatment with antimonials. Infections caused by *L. mexicana* evolve slower, initially resolve without therapy, but often recur despite treatment [7, 8]. Living with an active ulcer often involves constant pain, co-infections, community rejection due to stigma [2], decreased ability to work, and thus deterioration of the family economy. In turn, healed CL ulcers leave lifelong scars and may cause disfigurement and chronic disability [1, 9].

Official CL control efforts in Guatemala started in 2003, when the Ministry of Health (MoH) created the national control sub-program for CL (in Spanish, *Subprograma de Leishmaniasis*) as part of the wider Vector-Borne Disease Program. Current efforts focus on disease surveillance, diagnosis via tissue smear microscopy, and on providing free treatment to the affected individuals using injections of meglumine antimoniate (MA) [10]. Health education and vector control activities such as household insecticide spraying are also carried out in response to outbreaks [9]. The CL sub-program benefits from periodic financial and practical support from the Fundación Damián and the Universidad del Valle de Guatemala (UVG), mostly during outbreaks, and since 2014 has also received economic and technical support from the Pan American Health Organization (PAHO) [11].

According to data provided by SIGSA, in 2002 there were 1311 cases of CL at the national level. From 2003 to 2017 the average number of recorded cases was 674 per year, with high and low peaks (Additional file 1: Table S1). Reporting slightly different numbers, data from PAHO also indicates that there has been a large increase in CL cases over the last years, from 254 in 2014 to 775 in 2017 and 1044 in 2018, an increase of 117% in incidence rate from 2017 to 2018 [12]. Furthermore, over 1350 cases were reported by SIGSA in 2019. This increase in numbers could reflect surveillance improvements [13], but also illustrates shortcomings of current efforts. However, since the current program does not include monitoring and evaluation of strategies [9, 10, 14], the strengths and limitations of existing control efforts have not been critically evaluated. This study aimed to understand barriers and facilitators of CL control in the endemic Cobán municipality as experienced and perceived by key stakeholders in order to comprehend what works and does not. The identification of such perceived relations and their pathways can help develop evidence-informed interventions in the study region, and can assist wider NTD action in Guatemala and beyond.

## Methods

### Study setting

**Fig. 1:**
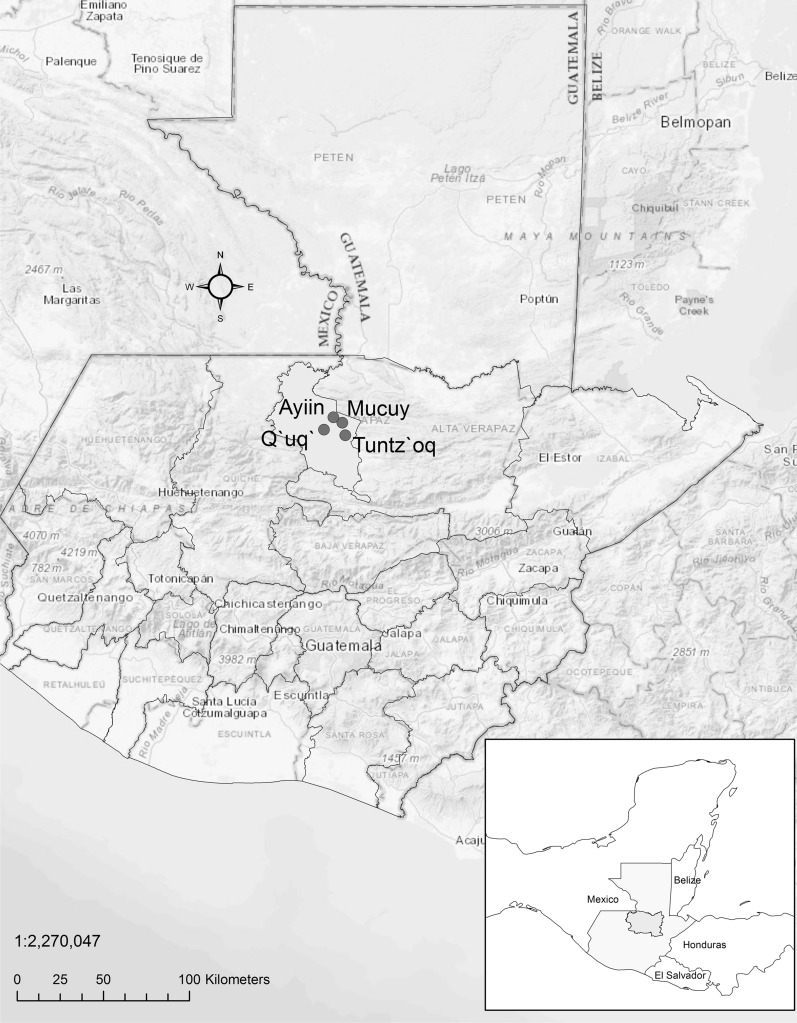
Study site: Alta Verapaz, Cobán municipality and selected communities

The study was conducted in the Cobán municipality, located in the department of Alta Verapaz, northern Guatemala (Fig. 1). According to the 2018 national census, Cobán has a population of 212 421 inhabitants, with an average age of 24.8 years [15]. Most of the population lives in urban areas (defined as having more than 2000 inhabitants, and electricity and piped water in 51% or more of the households [16]), and self-identifies as Mayan (85%)—mainly Q´eqchi´ (96%) [15]. Alta Verapaz is the poorest department in Guatemala, with 83.1% of the population living in poverty (having less than USD 5.5/day) and 53.6% in extreme poverty (less than USD 1.9/day) [17, 18]. As in other endemic regions of the country, the main economic activities are subsistence agriculture, livestock, hunting and forestry (19.6%), manufacture (12.6%) and small trade (34%) [17]. Literacy levels are 75.18%, but the net school attendance rate for children over seven years of age is only 31% [15].

The main causes of morbidity in Alta Verapaz are the typical of poverty-burdened populations: acute respiratory, skin and digestive infections [19]. There are only two doctors for every 10 000 inhabitants [20], well below the threshold of 44.5 per 10 000 inhabitants set by the World Health Organization [21]. It is estimated that the population without healthcare access at the community level is up to 72.4% [22]. This is the department with the most reported cases of CL in Guatemala (Additional file 1: Table S2), with over 60 400 people at risk of contracting CL only in Cobán according to MoH estimates [23].

For our study, we selected four communities with slightly different degrees of urbanisation, socio-economic development and healthcare access that had reported new CL cases during the 6 months preceding the study (Table 1).

**Table 1:** Living conditions and access to basic services in the endemic communities

Community*	Total population (2018)*	New CL cases reported in 2018 (% of total population)**	Electricity	Piped water	Roads/public transport	Telephone	Healthcare facilities (or distance to nearest)
Q´uq´	151	2 (1.3%)	No	No	Unpaved/taxi bikes	No	10.5 km
Ayiin	291	14 (4.8%)	Yes	No	Unpaved/No	Yes	21.6 km
Mucuy	191	14 (7.3%)	No	No	Unpaved/No	Yes	6.4 km
Tuntz´oq	1866	26 (1.4%)	Yes	No	Partially paved/minibus or taxi bikes	Yes	One primary care health post

*To protect informants’ identity we have replaced community names by fictitious ones

**CL Cutaneous leishmaniasis. Data from Ministry of Health registries for 2018

### Study design, sampling and recruitment

Data collection was based on focus groups and semi-structured interviews with healthcare providers, service users and potential users, including MoH officials, local health workers, people affected or at risk from the selected endemic communities, and community health workers (CHWs) informally involved in CL control (formally hired by the MoH until 2014 when the CL CHWs program was discontinued). Sampling was purposeful: potential informants were chosen based on their knowledge about current CL interventions or experience using CL control services, and because of their role in CL control.

To recruit residents of the endemic areas, we first approached community leaders, who then invited community members and informal community health workers (ICHWs) to participate as research informants in accordance with the procedures of each community. In the case of key MoH officials and local health workers, we recruited participants according to their availability for interview.

### Data collection and management

Data were collected during May and June 2019 by qualified researchers from the Center for Health Studies of the Universidad del Valle de Guatemala (CHS-UVG), with the support of a local native Q´eqchi´ speaker previously trained on obtaining informed consent and on the aims and methods of the study.

Semi-structured interviews with healthcare providers were conducted by CHS-UVG researchers in a place agreed with the participants. Held in Spanish, interviews were audio recorded when participants agreed. Otherwise, with their consent, researchers collected interview notes. During interviews, participants were asked about CL causes and treatment, local residents’ disease management, control measures implemented by the leishmaniasis program, the aspects of the program that in their experience worked well or could improve, and suggested potential solutions to existing problems. Background information regarding job position, and CL training was collected for each participant.

Focus groups with residents of the selected endemic communities were conducted by CHS-UVG researchers in community halls as agreed with community leaders. We conducted four separate focus groups, one in each community. Each focus group involved ten participants, all of them adults, five males and five females. Focus group data concentrated on participants’ understanding and management of CL, as well as on the strengths and limitations of the current CL interventions as perceived and experienced by the members of the community. Having endured years of internal armed conflict, people living in this region can be fearful and wary of outsiders. To respect their privacy and preserve their trust, in addition to anonymizing the names of the selected communities, no background information was pursued for this group of informants. Discussions were conducted in Q´eqchi´ and main topics and conclusions were annotated in paper boards while one researcher took parallel notes of the discussions in Spanish.

Data collected via focus groups and interviews was complemented by an examination of registries, normative and practical documents of the MoH at the national and local levels, mainly focusing on protocols, flowcharts and record keeping.

### Data analysis

Interviews and focus group discussions were first transcribed to text in Spanish by research assistants. The researchers who collected the information reviewed the transcripts and complemented these with their field notes. Together, all collected data was analyzed using a combination of thematic and content analysis through NVIVO 11 (QSR International, Massachusetts, USA) in order to identify good practices, challenges and potential points of CL control improvement in the region. To do this, we first conducted an inductive analysis to define the key issues repeatedly mentioned by informants. Three major themes emerged from data: resource scarcity, treatment-associated problems and knowledge-action gaps. We then scrutinized each theme via inductive content analysis in order to understand people’s particular experiences and the general dynamics within each theme. Minor disagreements were resolved through discussion.

### Research ethics

The study was approved by the National Ethics Committee of the Ministry of Public Health and Social Assistance of Guatemala (01-2019), the Research Ethics Committee of the CHS-UVG (191-02-2019), and the Ethics Review Committee of PAHO/WHO (PAHO-2019-02-0010). Accordingly, all research participants provided consent by signing or providing fingerprint in the presence of an impartial witness. A Q'eqchi´-Spanish translator trained on research ethics assisted researchers when needed. Codes were assigned to participants before any data was collected so the information cannot be traced back. The MoH researcher did not participate in data collection or analysis so as not to affect the opinions of health or community workers regarding the program or the conclusions of the study.

Study results were communicated to research participants in two separate meetings, one with healthcare providers and another with representatives of the four communities that participated in the study. During these final meetings research participants informally validated research results.

## Results

### Characteristics of research participants

From a total of 67 research participants, 27 (40.3%) healthcare providers were interviewed, and 40 (59.7%) residents of the endemic area participated in focus groups (Table 2).

**Table 2:** Categories and subcategories of informants

Cutaneous leishmaniasis healthcare providers (27*)	MoH officials (4)	National level (Guatemala) (3) Regional level (Alta Verapaz) (1)
	Local health workers (18)	Nurses (1), doctors (1), Vector control specialists (12), Healthcare assistants (4)
	Informal Community Health Workers (ICHWs) (5)	
Residents of endemic communities (40)	Q´uq´ (10), Ayiin (10), Mucuy (10), Tuntz´oq (10)	

(*) Number of informants in each category and sub-category

All interviewed healthcare providers combine CL control alongside various other vector-borne diseases activities. Interviewed local health workers were mostly male (81%), and had between 15 days and 26 years of experience. In contrast, ICHWs where mostly female (60%), with 3–30 years of experience taking it to themselves to help people affected by CL by administering MA injections and facilitating pain management using local plants.

**Fig. 2:**
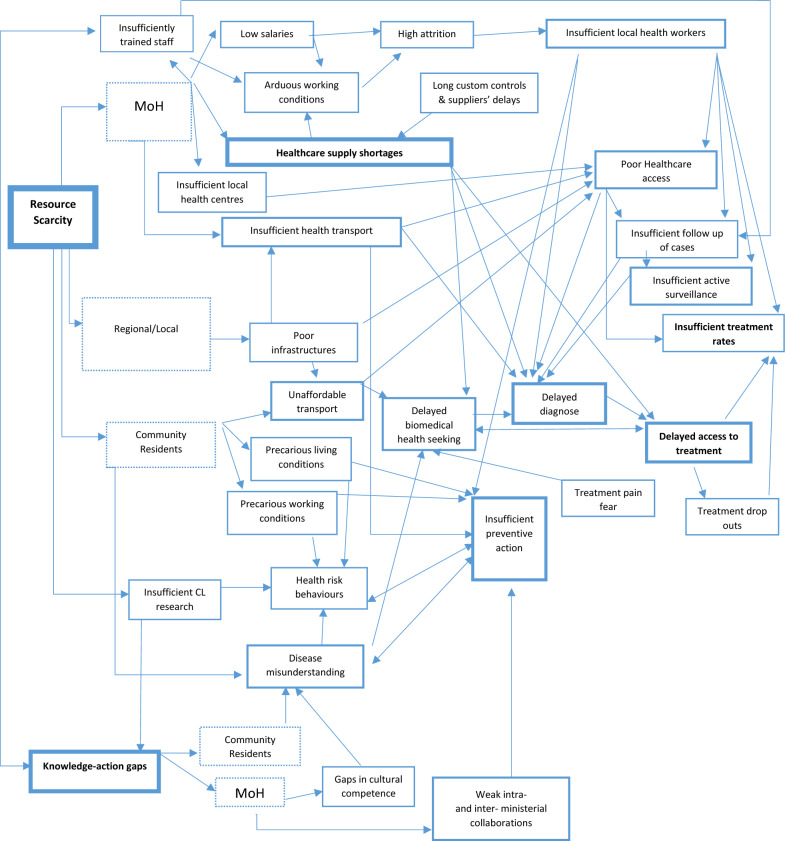
Aggregated mental model of problems associated with cutaneous leishmaniasis control and their causal relations as discussed by informants. (The width of the borders reflects the number of mentions). *CL* Cutaneous leishmaniasis, *MoH* Ministry of Health

Data analysis highlights three main and interrelated issues that help explain why CL control in Guatemala has stalled despite continued efforts: a) resource scarcity, b) treatment-associated challenges, and c) knowledge-action gaps (Fig. 2).

### The underlying challenge to CL control: resource scarcity

Resource scarcity is the core barrier to CL control in Cobán (Fig. 2). It translates into insufficient and poorly trained staff, healthcare supply shortages, and local health workers’ inability to travel to the local communities to promote prevention, search for new cases, and treat known ones.

Workers’ low salaries (USD 450/month, slightly above the minimum national wage) difficult recruitment. Combined with arduous working conditions and insufficient training, attrition rates are high. The resulting chronic shortages of qualified personnel negatively affect CL prevention, diagnosis, treatment and surveillance. Scarce economic resources also limit availability of vehicles to ensure access to the communities, forcing local health workers to walk long distances or use local transportation: ‘… we get there (Mucuy) by foot, which is a 45 min’ walk… If we pay a *tuc tuc* (motorized tricycle) it’s GTQ15 (USD 2), and that’s an extra expense we cover from our own pocket’. The MoH reimburses these expenses, but the process may take several months. The same occurs with healthcare supplies: ‘Sometimes when nurses don’t have needles… they buy from their own pocket’, explained another local health worker. Local health workers recognize that road improvement would facilitate their work. Aware that the allocation of funds among ministries and regions go beyond the scope of the MoH and CL sub-program, they identified motorcycles as a cost-effective solution.

Unaffordable economic costs, mobility issues, care for dependents, and work obligations also limit community residents’ ability to reach the health centres. As a Q´uq´ woman said: ‘public transport costs GTQ 40 (USD 5), and on top we lose a day of work’. For them, the most effective aspect of current CL interventions are health workers’ visits to their communities. During these trips, health workers actively search for new cases, treat known cases, and tell residents how to prevent bites and treat the ulcers. ‘If it wasn’t for them my daughter wouldn’t have healed’, said a woman from Ayiin community.

However, due to economic scarcity health professionals’ trips to the affected communities happen mostly during vector-borne diseases outbreaks. In addition, when asked about priority-setting and about balancing the scarce resources dedicated to controlling various diseases simultaneously, one experienced vector control specialist admitted that ‘cutaneous leishmaniasis is not our priority’. Active surveillance is not specific for CL, and priority is given to managing local diseases with higher burden and incidence such as dengue and malaria. ‘CL cases are often only randomly identified’, said another health worker. According to 2017 data from the Cobán health registry, 154 cases were identified because of community resident’s trips to the health centers (passive surveillance), and no cases were identified though active surveillance. In 2018, 156 cases (81%) were identified via passive surveillance.

### Treatment-associated challenges to CL control

Community residents agree that treating CL early with *“las ampollas”* (as MA vials are called in Cobán) avoids great pain. Limited healthcare access, fear to the painful injections of MA, and reluctance to show CL ulcers due to feelings of shame (expressed in Spanish as *“vergüenza de la enfermedad”)* however result in people using first local remedies, and they typically postpone the help of biomedical health professionals until they have exhausted community-based management options. For local health workers, the older the lesion the more difficult it is to detect parasites in tissue samples, which results in false negatives. When people finally seek medical attention and a full diagnosis has been possible, ulcers are often more difficult to treat. In Cobán, community-based treatments often involve applying battery liquid, nail polish, plant-based ointments, chili, amoxicillin, hot ashes, or gunpowder directly on the ulcers. Informants also reported the application of small doses of MA, illegally sold without prescription at pharmacies, injected directly in the ulcer.

There are also important glitches in the actual procurement, delivery, and administration of treatment. According to MoH guidelines, CL treatment should be administered shortly after diagnosis, and involves daily intramuscular injections for twenty consecutive days. Due to its toxicity and potential secondary effects, MA must be administered by health professionals inside a healthcare facility [9]. In practice, however, because of the above-mentioned transportation problems, local health workers provide individuals with ten vials and ask them to return to the health center for more to complete treatment. Furthermore, according to various community residents and local health workers, treatment waiting times can range from 6 to 12 months. A nurse explained that delays in drug supplies at national and local level often result in low adherence and treatment dropouts: ‘this waiting time makes patients feel not appreciated by the health system, including the staff, which often causes people to refuse treatment’.

For community residents however the problem with CL treatment is about the uncertainty regarding when and where the next treatment will be available. For many, going to a healthcare facility means ‘uncertainty’, ‘slow attention and diagnosis’, ‘incomplete treatments’ and, ultimately, ‘not finding a solution to their health problem’. As a Q´uq´ man said: ‘We go to the health services to waste our time because there’s no medicine…’. MA procurement is managed centrally in yearly bulk purchases that often incur delays associated with suppliers’ late deliveries and custom controls. According to 2017 data from the Cobán health registry, 24 people diagnosed with CL (16%) did not complete treatment, whilst only 24 people received treatment in 2016 (25%). Sixty-four people (80%) completed treatment in 2015. These delays also influence people’s reluctance to seek the advice from health professionals*.*

### Knowledge-action gaps obstructing CL control

Predominantly impeding preventive action are knowledge needs regarding the vector of the disease. Several community residents admitted not knowing how the disease was contracted. Similarly, experienced health workers have noticed that women take twice as long as men to seek help (12 months on average) and a higher CL prevalence among young males (15–24 years) − 22.6% of cases according to local registries. The particular dynamics that explain these differences are however not known, meaning little can be done in terms of preventive action. This is particularly important because pregnant women cannot receive MA injections. Talking about the most vulnerable groups and delayed health-seeking, healthcare workers mentioned the importance of thermotherapy as treatment option. MoH officials admitted nationwide plans to include thermotherapy in CL guidelines, but progress has been slow.

Organizational communication gaps are also hindering CL control. Besides having no clear information (or decision-making power) regarding healthcare supplies, several local health workers admitted they had never seen existing guidelines for CL control. Out of 18 health workers, 7 (38%) admitted never having received CL training, and 4 (22%) had only received one general tutorial. These workers admitted needing CL sample collection and treatment training, and guidance on how to follow-up false negative cases. MoH officials are aware of the ongoing staff training needs but lack resources to organize frequent trainings. Cost-effective, paper-based training materials have never been developed.

There are also inter-cultural communication gaps. Local health workers admitted knowing very little about people’s understandings of the disease, or about how to integrate biomedical treatment with local beliefs. Yet, the understanding of local beliefs and practices are not perceived as a learning need, whilst training community residents on biomedical understanding of disease causation and treatment appear as a key behavior change strategy in various interviews and focus groups. Community residents often associate CL with working in the forest, the presence of garbage around the house and dog ulcers. Several believe that it can be transmitted from person to person, and some attributed CL to ‘having seen something improper’. There are however no scheduled events dedicated to inform community residents about CL causes and treatment options. Furthermore, the MoH issues educational resources only in Spanish. No educational campaigns in the local language have ever been developed.

Addressing CL control challenges requires external collaborations, but these incur important barriers. Increasing coordination between those controlling healthcare stock at the local level and those purchasing it at the national level could help overcome stock shortages, but little can be done to ensure suppliers meet delivery times. Similarly, complex bureaucracies within and between ministries have impeded educational collaborations: ‘We even developed teaching materials, but local schools focused on immuno-preventable diseases’, explained a MoH official.

CL is a poverty-driven disease caused by scarce resources at national and local levels and its multiplicative impacts on peoples’ living conditions and on current control efforts. Income-generating interventions are however not perceived as strategic control avenues. For healthcare providers, the uneven distribution of national funds and scarce programmatic resources are at the core of the challenges faced by current CL efforts in Cobán. They identified the inclusion of formalized CHWs as a sustainable solution. The MoH had involved CHWs in the past, but this was discontinued in 2014 due to disagreements with the contracts. Research participants agree that the involvement of CHWs helped prevent key CL control challenges, which seems to be supported by PAHO data showing a significant increase in CL incidence nationwide since 2014[12]. Former CHWs continue to do this role informally without access to training or healthcare goods. There are no plans to formally restore their involvement.

## Discussion

The stagnation of CL control is tied to historic socio-economic marginalization of the study region and populations at risk which, among other unmet basic needs, live with chronically poor infrastructures and limited healthcare access. It is also tied to a chronic precariousness of the health system, characterized by insufficiently trained staff and resource shortages that hamper prevention activities and both surveillance and treatment rates. The Guatemalan Vector-Borne Diseases Program combines existing health resources to tackle malaria, Chagas, dengue, onchocerciasis, leishmaniasis and other arboviral diseases [14]. This integrated strategy, promoted worldwide by the WHO to optimise the use of limited resources, has been effective for instance in Bangladesh, India, Nepal, Ecuador and Colombia, where several vector-borne diseases also coexist [24–26]. Research indicates that key local socio-cultural factors such as people’s disease perceptions and financial accessibility determine disparities in the effectiveness of this integrated approach to vector disease management [24]. Our study shows that, in Guatemala, it is primarily the lack of funds dedicated to the program and to local socio-economic development what explains the lack of progress in CL control. CL is clearly a poverty-driven disease that socio-economic development can help prevent [1, 27]. In turn, as shown, programmatic resource scarcity underlies all CL control challenges, simultaneously shaping prevention, surveillance, treatment, and knowledge-action gaps despite the individual commitment and effort of some service providers.

Research indicates that programmatic resource scarcity obstructs integrated vector management because it limits preventive efforts, capacity building and evidence-informed decision-making [28–31]. In Bangladesh for instance, resource scarcity and weak logistics obstructed visceral leishmaniasis control efforts within an integrated vector management strategy by preventing regular vector surveillance and efficient preventive action [30]. Our research shows that the limited progress in CL control in Cobán is also significantly determined by a lack of rigorous decision-making when it comes to deciding what specific resources and efforts are dedicated to managing CL. As shown, CL control is not the main concern of local health workers. Prioritizing efforts towards the management of other local diseases with higher burden, CL mostly receives attention during outbreaks, and routine vector control activities are rare or nonexistent. The problem is however that the exact magnitude of CL is not really known, and neither is the socio-economic impact of the disease. Yet these indicators are needed for evidence-informed decision-making in terms of distribution of actions and allocation of funds within this integrated strategy. As shown, according to local registries 80% of CL cases were recorded via passive surveillance in 2018, and 100% in 2017 since no active surveillance was conducted during that year. However, passive surveillance is known to significantly underestimate incidence [32]. Our research also indicates that existing data – the 1357 cases reported for 2019 – is an underestimation, and real numbers are likely to be much higher. More research is needed to assess the real magnitude and burden of the disease in order to better prioritize actions and allocate reasonable funds.

The increase in CL incidence over the last years could partially reflect surveillance improvements [13]. It also indicates significant failures in prevention efforts. Our research highlights knowledge gaps that obstruct preventive action. In particular, regarding CL transmission cycles, vector and reservoir species incrimination, and the particular behaviours that explain different incidence rates by age and gender. Having this information is key to reinforce efforts, but this is impeded by the limited resources available within the CL program and dedicated to researching NTDs more generally.

Extensive literature illustrates the various ways in which resource scarcity influence the low success of NTD interventions [31, 33–35]. Our research indicates that socio-economic marginalization at the local level influences high incidence rates in that people continues to live in such levels of poverty that puts them at risk whilst due to an underfunded health program the effectiveness of control efforts is limited. Entomological and behavioral social research aimed at understanding differences in health-seeking behaviors and CL incidence by age groups and gender, improved roads and infrastructures, and increased NTD control budgets are all needed to strengthen CL control in the study region. Smaller cost-effective actions can also significantly improve CL control outcomes, mainly by streamlining action using existing knowledge and assets.

First, the development of educational materials for both service providers and community residents is paramount. CL service providers simultaneously work on various vector-borne diseases and are not exclusively dedicated to controlling CL. This strategy to maximize scarce resources is not a problem per se, but becomes problematic when workers lack the necessary training. As shown, 60% of interviewed health workers had not received sufficient CL training. Interestingly, during focus groups it became evident that several community residents confused CL vector control measures with those of dengue (such as keeping clean the household and its surroundings to avoid the accumulation of water, breeding site of the *Aedes* mosquitoes). Confusion between coexisting vector-borne diseases transmitting vectors has also been reported in Colombia [36], where local population involvement in the design of interventions was recommended as a strategy to strengthen prevention. Unfortunately, we were not able to follow up whether this was just a problem of misunderstanding or due to errors in the information provided by local health workers, and this is a limitation of our study. Similarly, we could not explore the dynamics that might help explain the above-mentioned differences in prevalence and incidence rates by age groups and gender, which constitutes another limitation of our study. Nevertheless, the CL training needs of service providers is clear, and so is the need for culturally appropriate educational campaigns, either via the CL sub-program, radio adverts or collaborations with local schools [37].

Small changes in procurement and CL treatment can also have a positive impact on treatment and cure rates, and possibly also on the number of people seeking the advice of health professionals. NTD research demonstrates that trust towards health providers positively affect health-seeking decisions and the outcomes of public health interventions [38]. To consolidate (or re-establish) trust, existing literature recommends ensuring health workers are properly trained [31, 36], intercultural competence in disease control efforts [39, 40] and job permanency to guarantee long term relations between health providers and community residents among other [41]. Our research contributes to this body of literature by illustrating how, despite recognizing and appreciating the personal efforts made by some health workers, community resident’s lack of trust towards the Guatemalan health system often means that people affected by CL do not seek the advice of health professionals. In Cobán, community resident’s reservations towards healthcare providers are influenced by people’s fear of painful MA injections and its adverse reactions, awareness of treatment shortages, and uncertainty regarding treatment availability. As shown, these relationships often push community residents to seek ulcer management alternatives proven to aggravate the disease and difficult diagnosis, and ultimately to refuse treatment and avoid the help from health workers. This is important because, as the number of people diagnosed with CL is rising, qualitative information reveals that not all affected individuals receive timely treatment. Engaging trained (and paid) CHWs able to take diagnose samples and treat people may pose a sustainable solution [42]. Procurement optimization must follow. Current MoH protocols establish MA as the only drug to treat CL in Guatemala [9], but customs and procurement issues cause important delays and shortages at the local level. As shown, 84% of people diagnosed with CL in the study site completed treatment in 2017. In 2018, the MoH spent USD 80.00 per person on CL treatment and diagnosis (personal communication), USD 83 520 in total. This is a significant cost. Thermotherapy has been shown to be more cost-effective than MA to treat CL and to cause fewer side effects [43–45]. Alongside the engagement of community health workers, its incorporation can help address many of the challenges to current efforts, effectively strengthening surveillance and cure rates whilst reducing program costs.

Our study highlights not only the barriers to the successful management of CL in four endemic communities of the Cobán municipality, but also aspects of the current efforts that seem to work well. Some limitations should be recognised. To the above-mentioned limitations we would like to add that the research team spent only a few days collecting information. A longer interaction with research participants may yield different results. Also, although participants were diverse in terms of age, gender, occupation and place of residence, given the case-study approach of our research results may not be generalizable to other endemic contexts.

## Conclusions

The Guatemalan health system operates in a shortage crisis, with limited human resources and insufficient infrastructure. CL control challenges are tied to the system´s precariousness and are not isolated. Health personnel cover multiple first-level healthcare needs of a highly impoverished population living in regions that are endemic for various diseases, lacking the time, training and resources to properly control CL. More resources need to be allocated to this economically marginalised region to improve roads, access to basic services, people’s economic assets and health worker-population ratios among other. More research is also needed to develop evidence-informed preventive strategies. Smaller but cost-effective actions with great potential for strengthening CL control despite current fund allocation deficits are: the development of CL training materials for local workers and ensure workers’ access to existing guidelines; decentralise and smoothen MA procurement and systematise the use of thermotherapy; develop educational material in Q’eqchi´; provide health centres with motorcycles to strengthen active surveillance and treatment rates and reinstate the involvement of trained community health workers.

## Supplementary Information

**Additional file 1: Table S1.** New cases of Cutaneous leishmaniasis in Guatemala (2001-2019). **Table S2.** Incidence of Cutanous leishmaniasis in endemic departments (2019)*.

## Data Availability

All statistical data generated or analysed during this study are included in this published article and its supplementary information files. Fully anonymised and non-identifiable qualitative data has been stored in the ‘Brighton and Sussex Medical School (BSMS) Sharepoint’ and are available from the corresponding author on reasonable request.

## Abbreviations

CL: Cutaneous Leishmaniasis
INE: *Instituto Nacional de Estadística*
MA: Meglumine antimoniate
MoH: Ministry of Health
MSPAS: *Ministerio de Salud Pública y Asistencia Social*
NTD: Neglected Tropical Disease
PAHO: Pan American Health Organization
SIGSA: *Sistema de Información Gerencial de Salud*
WHO: World Health Organization
