# Supplementary material for: Cutaneous leishmaniasis control in Alta Verapaz (northern Guatemala): evaluating current efforts through stakeholders’ experiences
Source: Infect Dis Poverty. 2021 May 7;10:61. doi: 10.1186/s40249-021-00842-3 (PMC8106169; doi:10.1186/s40249-021-00842-3)
Supplement: Supplementary file 1 — Additional file 1: Table S1. New cases of Cutaneous leishmaniasis in Guatemala (2001-2019). Table S2. Incidence of Cutanous leishmaniasis in endemic departments (2019)*. [file 40249_2021_842_MOESM1_ESM.docx]

**Additional Table 1. New cases of Cutaneous Leishmaniasis in Guatemala (2001-2019)**

| **year** | **Total number of cases*** |
| --- | --- |
| 2001 | 527 |
| 2002 | 1311 |
| 2003 | 805 |
| 2004 | 827 |
| 2005 | 1323 |
| 2006 | 627 |
| 2007 | 326 |
| 2008 | 439 |
| 2009 | 332 |
| 2010 | 626 |
| 2011 | 463 |
| 2012 | 670 |
| 2013 | 675 |
| 2014 | 688 |
| 2015 | 769 |
| 2016 | 800 |
| 2017 | 740 |
| 2018 | 1001 |
| 2019 | 1357 |

(*) Data provided by SIGSA, the National Health Management Information System

**Additional Table 2. Incidence of Cutaneous Leishmaniasis in endemic departments (2019)*.**

|  | **Reported cases (total)** | **Population** | **Incidence (/100,000 inhabitants)** |
| --- | --- | --- | --- |
| **Guatemala** | 1357 | 4160144 | 32,62 |
| **Alta Verapaz** | 831 | 1149998 | **72,26** |
| **Baja Verapaz** | 1 | 11659 | 8,58 |
| **Escuintla** | 1 | 170543 | 0,59 |
| **Guatemala** | 3 | 995130 | 0,3 |
| **Huehuetenango** | 28 | 193625 | 14,46 |
| **Izabal** | 15 | 209988 | 7,14 |
| **Jalapa** | 1 | 181437 | 0,55 |
| **Petén** | 439 | 698187 | 62,88 |
| **Quetzaltenango** | 1 | 168880 | 0,59 |
| **Quiche** | 29 | 216573 | 13,39 |
| **San Marcos** | 1 | 76593 | 1,31 |
| **Santa Rosa** | 1 | 11641 | 8,59 |
| **Zacapa** | 6 | 75890 | 7,91 |

(*) Data provided by PAHO
